# Retroperitoneal Fibrosis Presenting With Duodenal Obstruction: A Fatal Case of Corticosteroid-Resistant Disease

**DOI:** 10.7759/cureus.103515

**Published:** 2026-02-13

**Authors:** Samuel J Roberts, Michael S Floyd Jr, Kaylie E Hughes

**Affiliations:** 1 Endourology and Reconstructive Urology, Whiston Hospital, Prescot, GBR

**Keywords:** duodenal obstruction, igg4-related disease, multidisciplinary management, retroperitoneal fibrosis, steroid-resistant disease

## Abstract

Retroperitoneal fibrosis is a fibro-inflammatory condition characterised by the development of dense tissue within the retroperitoneum, which may result in the compression of adjacent structures. We report the case of a 69-year-old woman with a history of breast cancer who initially presented with right-sided hydronephrosis. Cross-sectional imaging demonstrated retroperitoneal soft tissue encasing the ureters, consistent with retroperitoneal fibrosis. She underwent unilateral ureteric stenting followed by bilateral nephrostomy insertion and was commenced on corticosteroid therapy. Following review at a national specialist centre, a variant of idiopathic retroperitoneal fibrosis was diagnosed.

She was subsequently re-admitted with progressive complications including deep vein thrombosis, nephrostomy site bleeding, and *Staphylococcus epidermidis* bacteraemia. During this admission, the patient developed progressive upper gastrointestinal symptoms, including early satiety and vomiting. Computed tomography demonstrated gastric and proximal duodenal dilatation with tethering of the distal duodenum, consistent with duodenal obstruction. Endoscopy confirmed external compression at the third part of the duodenum. Despite escalation to high-dose intravenous corticosteroids and supportive management, her condition deteriorated with refractory vomiting, electrolyte imbalance, fluid overload, and respiratory compromise. She was deemed unsuitable for surgical or endoscopic intervention and was transitioned to palliative care, dying eight months after her initial presentation.

This case demonstrates a challenging clinical course of retroperitoneal fibrosis complicated by duodenal obstruction and highlights the complexities of diagnosis and management when extra-urological involvement develops.

## Introduction

Retroperitoneal fibrosis (RPF) is a rare fibro-inflammatory disorder characterised by the development of fibrosis in the retroperitoneal space [1,2]. The retroperitoneum is the anatomical space posterior to the peritoneal cavity and contains structures including the kidneys, ureters, aorta, and duodenum. In RPF, chronic fibro-inflammatory tissue proliferation within this space leads to progressive encasement and the compression of adjacent structures, most commonly the ureters. This frequently presents as obstructive uropathy. Rarely, the disease can involve the gastrointestinal tract [3-9].

The disease entity is broadly classified into two forms. The idiopathic form is thought to be part of the immunoglobulin G4 (IgG4) disease spectrum and makes up two-thirds of cases [2,10-12]. Secondary RPF is associated with malignancy, radiation, surgery, infection, or certain drugs such as ergot derivative medications, beta blockers, dopamine agonists, and hydralazine [2,10]. Here, we describe a case of idiopathic RPF causing the external compression of the duodenum that was poorly responsive to corticosteroid therapy, ultimately proving fatal.

## Case presentation

Initial presentation 

A 69-year-old woman presented to a district general hospital in the UK with right flank pain and unintentional weight loss. Her medical history included hormone receptor-positive breast cancer treated eight years previously with lumpectomy, radiotherapy, and chemotherapy. She also had a history of ventricular tachycardia complicated by cardiac arrest, for which an implantable cardioverter-defibrillator had been inserted.

Initial laboratory investigations demonstrated mildly elevated inflammatory markers with preserved renal function (Table 1). A computed tomography (CT) urogram demonstrated right-sided hydronephrosis with peri-ureteric soft tissue thickening. Subsequent ureteroscopy identified no intrinsic cause for obstruction, and a right-sided ureteric (JJ) stent was inserted. The patient was discharged with outpatient follow-up. 

**Table 1:** Initial laboratory investigations Na: sodium; K: potassium; eGFR: estimated glomerular filtration rate; WCC: white cell count; Hb: hemoglobin; MCV: mean corpuscular volume; CRP: C-reactive protein

Laboratory parameter	Value	Units	Reference range
Na	138	mmol/L	135-145
K	4.3	mmol/L	3.5-5.0
Urea	5.1	mmol/L	2.5-7.8
Creatinine	74	µmol/L	45-90
eGFR	71	mL/min/1.73 m²	>60
WCC	8.4	×10⁹/L	4.0-11.0
Hb	134	g/L	115-160
MCV	90	fL	80-100
Platelet count	234	×10⁹/L	150-400
CRP	20	mg/L	<5

**Figure 1:**
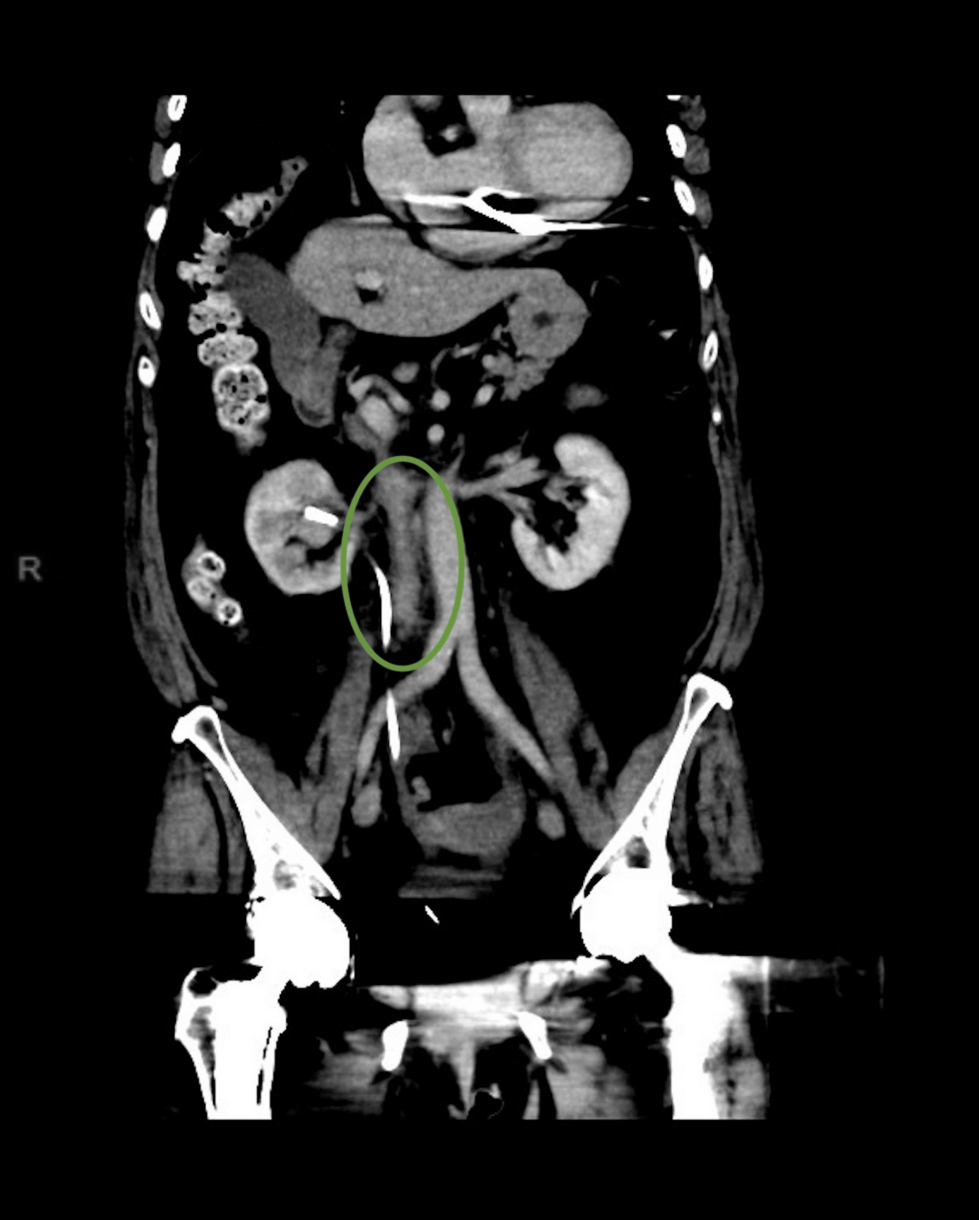
CT of the abdomen and pelvis (coronal view) showing an ill-defined soft tissue mass, predominantly right-sided, extending from the renal hilum to the aortic bifurcation CT: computed tomography

On follow-up imaging two months later, a repeat CT of the abdomen and pelvis demonstrated an ill-defined retroperitoneal soft tissue mass encasing the mid-ureter (Figure 1). Image-guided biopsy was considered but deemed not feasible due to the anatomical location. The radiological appearances were considered most consistent with RPF.

The following month, the patient developed new left-sided hydronephrosis and was admitted for bilateral nephrostomy insertion. At the time of this admission, renal function had deteriorated markedly, with an estimated glomerular filtration rate of 6 mL/min/1.73 m². Rheumatological investigations including inflammatory markers, immunoglobulin profiling, and IgG4 subclass testing were performed and were within normal limits (Table 2).

**Table 2:** Rheumatological laboratory investigations at nephrostomy admission ESR: erythrocyte sedimentation rate; IgA: immunoglobulin A; IgG: immunoglobulin G; IgG4: immunoglobulin G4; IgM: immunoglobulin M; ANA: antinuclear antibody; ANCA: anti-neutrophil cytoplasmic antibody

Laboratory parameter	Value	Units	Reference range
ESR	10	mm/hr	0-20
IgA	3	g/L	0.4-3.5
IgG	9.4	g/L	6.5-16.0
IgG4	0.097	g/L	0.03-2.0
IgM	1.04	g/L	0.5-3.0
ANA	Negative	-	Negative
ANCA	Negative	-	Negative

**Figure 2:**
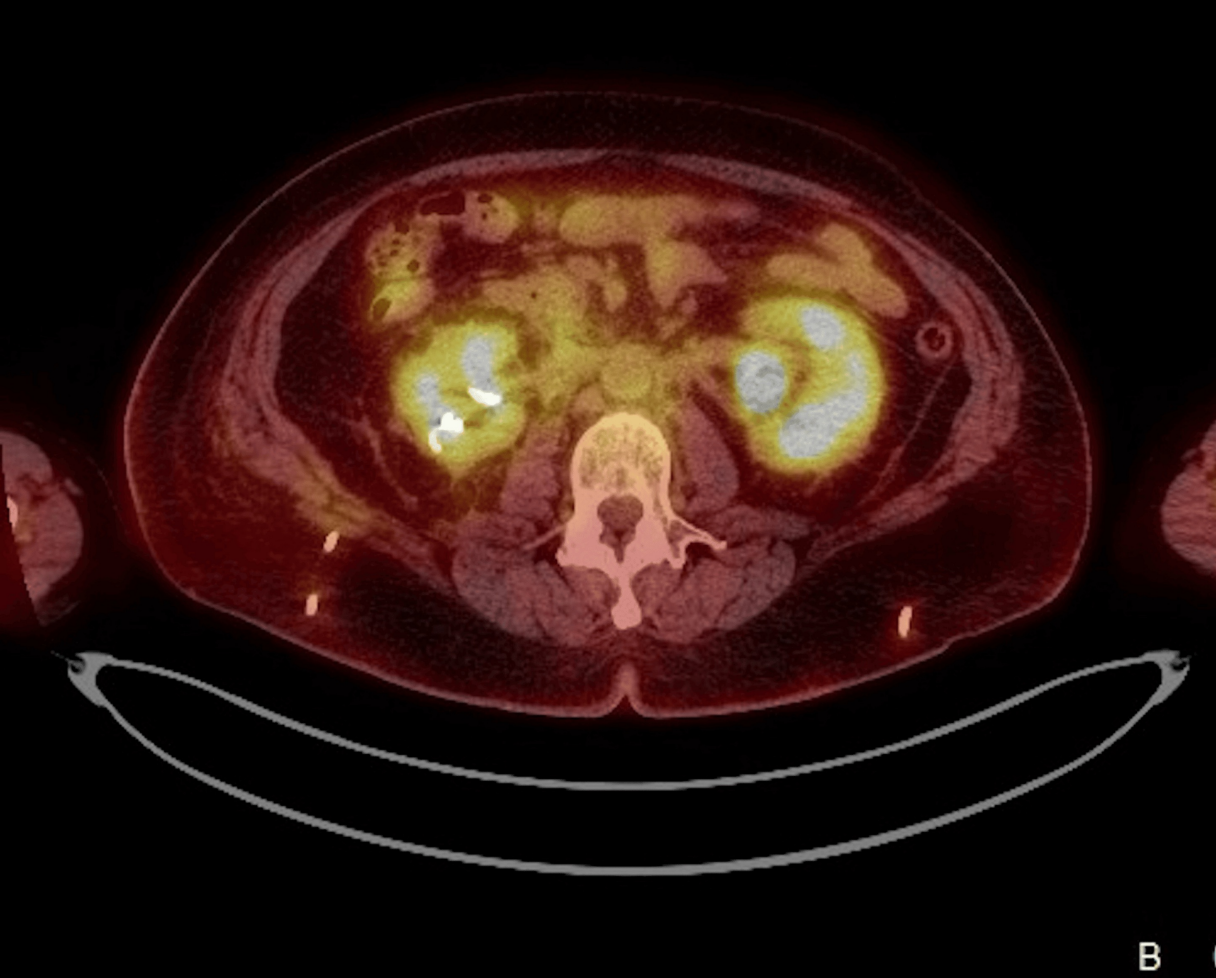
PET-CT (axial view) showing an ill-defined retroperitoneal soft tissue with mild increase in FDG activity PET-CT: positron emission tomography-computed tomography; FDG: fluorodeoxyglucose

An outpatient positron emission tomography-CT (PET-CT) scan showed no evidence of malignancy but demonstrated mild retroperitoneal inflammatory changes without aortitis or peri-aortitis (Figure 2). Following rheumatology review, she was commenced on oral prednisolone 50 mg daily.

Re-admission 

Six months after her initial presentation, the patient was diagnosed with a deep vein thrombosis and commenced on therapeutic anticoagulation before being discharged. She later presented to the hospital with bleeding from the left nephrostomy site requiring blood transfusion and was re-admitted. Bilateral nephrostomy exchange was performed, and anticoagulation was temporarily withheld.

**Figure 3:**
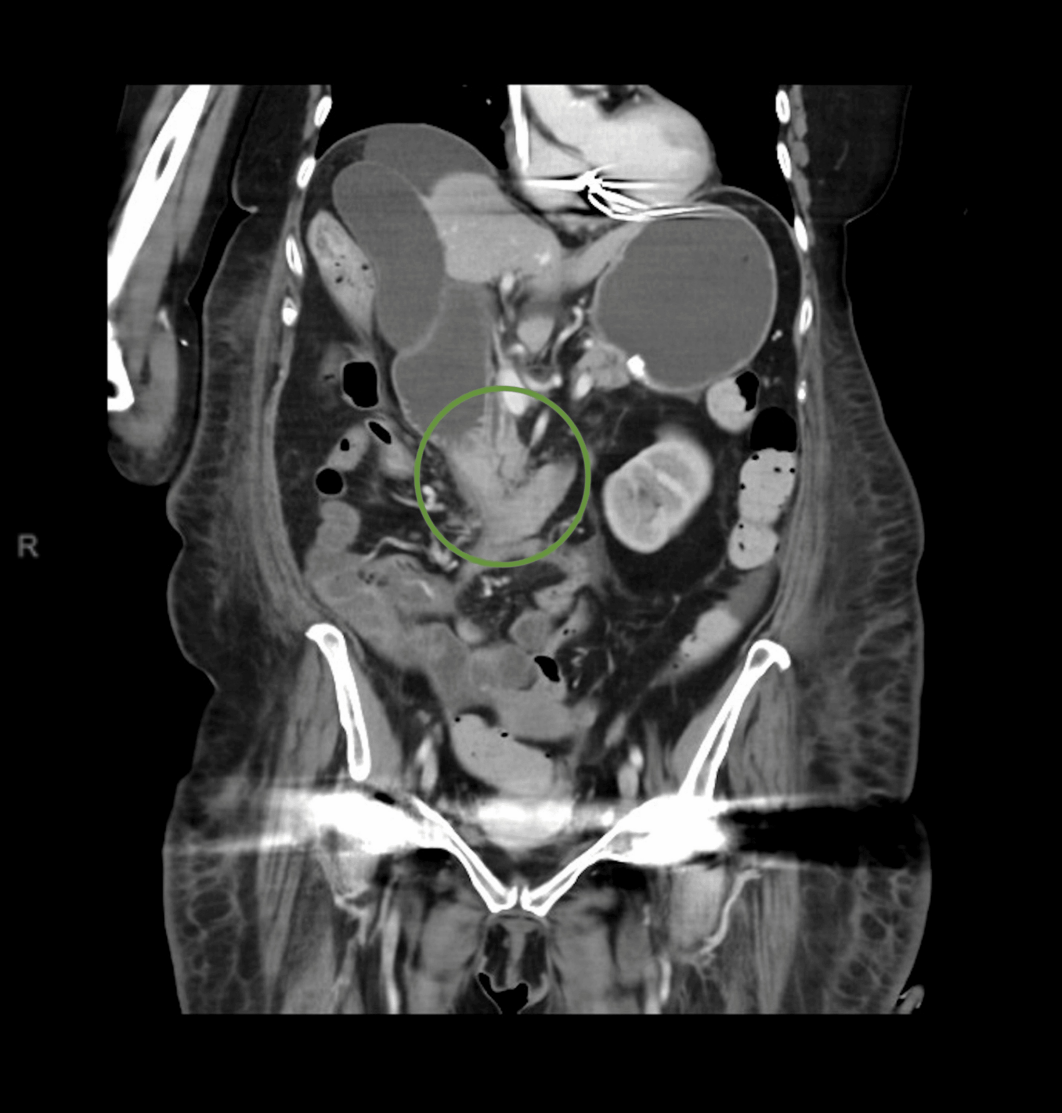
CT of the abdomen and pelvis (coronal view) showing the marked distension of the stomach and proximal D2 duodenum, with collapsed D2/D3, which was tethered secondary to retroperitoneal fibrosis CT: computed tomography

Her inpatient course was complicated by persistent *Staphylococcus epidermidis* bacteraemia, raising concern for an implantable cardioverter-defibrillator pocket infection. This was managed with intravenous teicoplanin and oral rifampicin. During this admission, she developed progressive gastrointestinal symptoms, including early satiety, vomiting, and further weight loss. A repeat CT of the abdomen and pelvis demonstrated marked gastric and proximal duodenal dilatation with collapse and tethering of the distal duodenum towards the RPF, consistent with duodenal obstruction (Figure 3).

Her condition deteriorated with persistent vomiting and inability to tolerate oral intake. Oesophagogastroduodenoscopy demonstrated external compression at the third part of the duodenum with failure of luminal distension despite insufflation. A nasojejunal feeding tube was passed beyond the obstruction for enteral nutrition. A barium swallow study was not tolerated.

The case was re-discussed with the national retroperitoneal fibrosis centre, and a trial of high-dose intravenous hydrocortisone (100 mg four times daily) was recommended to relieve the obstruction. A nasogastric tube was placed proximal to the obstruction for decompression and left on free drainage for symptom control. A repeat PET-CT scan demonstrated no evidence of endocarditis or device infection but again showed ill-defined retroperitoneal and mesenteric soft tissue thickening.

Despite maximal medical therapy, the patient's condition continued to decline. She developed refractory hypokalaemia secondary to ongoing gastric losses, and intravenous replacement resulted in progressive fluid overload with worsening pleural effusions. She was assessed by critical care but was not considered suitable for central venous potassium replacement. Upper gastrointestinal surgical review determined that she was not fit for operative intervention. Rheumatology review concluded that she had reached the ceiling of medical therapy, with a poor response to high-dose corticosteroids. Given her continued deterioration and poor prognosis, the patient was transitioned to palliative care, dying eight months after her first presentation. 

## Discussion

Pathophysiology of primary and secondary RPF

RPF is characterised by the development of fibro-inflammatory tissue in the retroperitoneum, typically surrounding the abdominal aorta and iliac vessels. This tissue can encase structures such as the ureters, inferior vena cava, psoas muscles, and lymphatic vessels and may extend as far caudally to include the gonadal vessels and spermatic cord. In rare instances, the duodenum and pancreatic head can also be involved [1,2].

RPF is classified into primary (idiopathic) and secondary forms. Primary RPF accounts for approximately two-thirds of cases, and many are now recognised as part of the IgG4-related disease spectrum [2,10-12]. Histological hallmarks of IgG4-related disease include storiform fibrosis, lymphoplasmacytic infiltration, and obliterative phlebitis, with or without elevated serum IgG4 levels [11,12].

Secondary RPF may arise in association with malignancy (particularly lymphoma or sarcoma), infection (e.g., tuberculosis), prior surgery, radiotherapy, abdominal aortic aneurysms, or certain medications such as methysergide, ergotamine, cabergoline, hydralazine, bromocriptine, and beta blockers [2,10]. 

Table 3 lists the primary and secondary causes of RPF.

**Table 3:** Primary and secondary causes of retroperitoneal fibrosis RPF: retroperitoneal fibrosis; IgG4: immunoglobulin G4

Feature	Primary (idiopathic) RPF	Secondary RPF
Underlying cause	Immune-mediated fibro-inflammatory process	Malignancy, drugs, infection, radiation, surgery
IgG4 association	May be present	Typically absent
Distribution	Often peri-aortic, ureteric involvement	Variable, may be focal or asymmetric
Response to steroids	Often responsive	Variable, often limited
Management focus	Immunosuppression ± stenting	Treat the underlying cause

Reported cases of duodenal involvement

Gastrointestinal involvement in RPF is uncommon. Since the 1960s, a paucity of cases describing duodenal obstruction has been published [3-9]. In the reported cases, duodenal obstruction was typically caused by the external compression of the second or third part of the duodenum by fibro-inflammatory retroperitoneal tissue, often in association with ureteric involvement. Most patients demonstrated symptomatic improvement following corticosteroid therapy, with surgical bypass or endoscopic intervention reserved for refractory cases [3-9,13].

Our case adds to the limited body of literature. In contrast to previous reports where corticosteroid therapy was often effective [3,4], our patient demonstrated a poor response to high-dose corticosteroids and was not a candidate for surgical intervention, ultimately with a fatal outcome. This case underlines the heterogeneous clinical course of RPF and the importance of considering duodenal involvement in patients with gastrointestinal symptoms. 

Symptoms of RPF

The most frequent presenting symptoms of RPF are flank or back pain, lower limb swelling, and constitutional features such as weight loss, anorexia, and fatigue [1,2,10]. Ureteric obstruction is common and may cause hydronephrosis and flank pain and lead to renal impairment [10].

When the duodenum is involved, symptoms are those of gastric outlet obstruction: early satiety, nausea, vomiting, abdominal distension, and weight loss [3-6]. These features were prominent in our case and were initially overshadowed by renal complications, highlighting the varied clinical manifestations of RPF.

Laboratory findings

Laboratory abnormalities are non-specific. Raised erythrocyte sedimentation rate (ESR) and C-reactive protein (CRP) are reported in up to 70% of cases but may be normal in others [2,10]. Renal impairment commonly reflects ureteric obstruction [5]. Serum IgG4 is useful when IgG4-related disease is suspected, but it lacks both sensitivity and specificity: up to 40% of affected patients may have normal levels [11,12]. In our case, inflammatory markers and serum IgG4 were normal, underscoring the limitations of serological testing in establishing a diagnosis.

Diagnosis

Cross-sectional imaging is key to diagnosis. CT typically shows a plaque-like mass encasing the aorta and ureters, often with medial ureteric deviation [10]. Magnetic resonance imaging (MRI) may better delineate disease activity, while PET-CT can help distinguish RPF from malignancy and guide biopsy [14,15]. Biopsy is not always technically possible but should be considered if imaging is atypical or malignancy is suspected [10,16]. Endoscopy has a role when there is gastrointestinal involvement, as in this patient, where it confirmed the external compression of the duodenum at the D2/D3 junction. 

Treatment

High-dose corticosteroids are the mainstay of treatment for idiopathic RPF, usually producing symptomatic and radiological improvement within weeks [2,10]. For refractory or relapsing disease, immunosuppressants such as azathioprine, mycophenolate mofetil, or methotrexate may be considered [2,10]. Rituximab has also shown benefit in IgG4-related RPF [2].

Mechanical obstruction may necessitate surgical intervention. Options include ureteric stenting or ureterolysis for urinary tract obstruction and duodenal stenting or surgical bypass for gastrointestinal obstruction [4-6,13]. Despite escalation to high-dose systemic corticosteroid therapy, the patient did not demonstrate clinical or radiological improvement, with progressive gastrointestinal obstruction and overall clinical deterioration. The patient did not tolerate endoscopic or contrast-based procedures, was unfit for surgery, and failed to respond to high-dose corticosteroids.

Alternative strategies such as the earlier consideration of steroid-sparing immunosuppressive agents or earlier multidisciplinary surgical evaluation may be discussed in similar cases. However, in this patient, severe comorbidity, advanced disease at presentation, and rapid clinical deterioration significantly limited available therapeutic options. This highlights the importance of the early recognition of atypical gastrointestinal manifestations of RPF and prompt referral to specialist centres, as earlier intervention may offer greater therapeutic flexibility in selected patients.

## Conclusions

Our case discusses a steroid-resistant variant of idiopathic RPF causing progressive and eventually fatal duodenal obstruction. This case demonstrates three critical lessons: first, that RPF can extend beyond the urinary tract to cause life-threatening gastrointestinal compression; second, that normal inflammatory markers or serum IgG4 levels do not exclude active disease; and third, that early multidisciplinary collaboration is essential to recognise atypical manifestations, assess for bespoke surgical options before a functional decline, and tailor immunosuppressive therapy.

Ultimately, this report expands the limited literature on gastrointestinal involvement in RPF and highlights the urgent need for greater awareness and research into steroid-refractory disease variants.
